# Determinação do posicionamento da linha articular do joelho por meio das distâncias bicondilar femoral e epicondilar na população brasileira

**DOI:** 10.1055/s-0045-1811629

**Published:** 2025-11-04

**Authors:** Leonardo Augusto Melo de Andrade, Márcio de Castro Ferreira, Carlos Eduardo da Siveira Franciozi, Enzo Salviato Mameri, Marcelo Seiji Kubota, Marcus Vinícius Malheiros Luzo

**Affiliations:** 1 Universidade Federal de São Paulo, São Paulo, SP, Brasil; 2 Grupo de Cirurgia do Joelho do Departamento de Ortopedia e Traumatologia da Escola Paulista de Medicina, Universidade Federal de São Paulo, São Paulo, SP, Brasil; 3 HCor, São Paulo, SP, Brasil

**Keywords:** artroplastia do joelho, osteoartrite, procedimentos ortopédicos, prótese de joelho, próteses e implantes, arthroplasty, replacement, knee, knee prosthesis, osteoarthritis, orthopedic procedures, prostheses and implants

## Abstract

**Objetivo:**

Determinar o posicionamento da altura da linha articular de joelhos a partir das referências anatômicas da distância do eixo bicortical condilar femoral e das distâncias epicondilares medial e lateral na população brasileira.

**Métodos:**

Foram analisados 500 exames de ressonância magnética de joelhos de 250 mulheres e 250 homens submetidos às mensurações das medidas do eixo bicortical condilar (EBC), da distância da altura articular do epicôndilo medial (DEM) e da distância do epicôndilo lateral (DEL).

**Resultados:**

A média de idade dos pacientes analisados foi de 50,91 ± 14,76 anos. A distância média do EBC foi 72,11 ± 5,93mm. As DEMs e DELs médias foram 33,39 ± 3,50mm e 26,32 ± 4,08mm. As fórmulas encontradas para estimar a distância da linha articular a partir do epicôndilo medial e lateral nos homens foram DEM = 0,4618 x EBC e DEL = 0,3615 x EBC e, nas mulheres, DEM = 0,4653 x EBC e DEL = 0,3767 x EBC para intervalo de confiança de 95%.

**Conclusão:**

A distância do EBC femoral pode ser usada como referência para a determinação do posicionamento da linha articular a partir das distâncias dos epicôndilos femorais medial e lateral.

## Abstract (en)

**Objective:**

To determine the positioning of the knee joint line height based on anatomical references, that is, the distance of the femoral condylar bicortical axis and the medial and lateral epicondylar distances in the Brazilian population.

**Methods:**

We analyzed 500 magnetic resonance imaging tests of the knees of 250 women and 250 men to measure the condylar bicortical axis (CBA) and the distance of the joint height to the medial epicondyle (MED) and to the lateral epicondyle (LED).

**Results:**

The mean age of the patients was 50.91 years old, with a standard deviation (SD) of ± 14.76. The mean CBA distance was 72.11 ± 5.93mm. The mean MED and LED values were 33.39 ± 3.50mm and 26.32 ± 4.08mm, respectively. The formulas to estimate the distance of the joint line from the medial and lateral epicondyles were MED = 0.4618 x CBA and LED = 0.3615 x CBA for male subjects and MED = 0.4653 x CBA and LED = 0.3767 x CBA for female subjects using a 95% confidence interval.

**Conclusion:**

The distance to the femoral CBA can be a reference for determining the joint line positioning from the distances of the medial and lateral femoral epicondyles.

## Introdução

A artroplastia total de joelho (ATJ) é a cirurgia padrão-ouro para o tratamento da gonartrose em pacientes que não respondem ao tratamento medicamentoso e fisioterápico. É um procedimento em crescimento mundial 1 2 e, ao mesmo tempo que as ATJ primárias aumentam, as taxas de revisões de artroplastias também acompanham o crescimento. Nos Estados Unidos, as revisões de ATJ correspondem a ∼ 10% dos números de artroplastias primárias. 1

Revisões de ATJ são cirurgias desafiadoras para os cirurgiões devido à complexidade provocada pela perda óssea associada às insuficiências ligamentares. Uma dificuldade primária é a determinação da altura da linha articular (LA) diante das perdas ósseas existentes. Restaurar o posicionamento da LA fisiológica é um dos princípios importantes na ATJ de revisão para melhorar a amplitude de movimento, otimizar o funcionamento do mecanismo extensor e manter a cinemática normal do joelho. 3

Muitos autores estudaram métodos estimados para definir a LA utilizando marcadores anatômicos como a cabeça da fíbula, a tuberosidade tibial e as distâncias epicondilares. 3 4 É preciso destacar que estudos em determinados grupos populacionais podem divergir em seus aspectos anatômicos do joelho de outros grupos étnicos. 5 6 7

Neste contexto, o objetivo do presente estudo foi analisar uma razão constante entre os marcadores anatômicos do joelho: distância do epicôndilo medial clínico (DEM), distância do epicôndilo lateral (DEL) e eixo bicortical condilar (EBC) para facilitar o cirurgião a estimar o posicionamento da linha articular em procedimentos de revisões de ATJ na população brasileira.

## Materiais e Métodos

O presente estudo foi aprovado pelo comitê de ética da Universidade Federal de São Paulo sob o número CAAE-83009724.5.0000.5505. Foram selecionados de maneira aleatória e anônima 500 exames de ressonância magnética (RM) de joelhos de 250 mulheres e 250 homens.

O critério de inclusão foi pacientes de ambos os sexos esqueleticamente maduros.

Os critérios de exclusão foram exames com deformidade morfológica óssea do joelho (osteófitos), exames com dispositivos médicos implantáveis e evidência de fratura articular prévia.

Os pacientes elegíveis foram identificados no banco de imagens de RM da instituição disponíveis em sua plataforma e no software Clinical Collaboration Platform (Carestream Health). No caso de pacientes com exames bilaterais, foi incluído para análise apenas o joelho direito.

As mensurações das DEM e DEL da linha articular femoral foram avaliadas em cortes coronais em sequência T2 e a distância do EBC foi mensurada em cortes axiais em sequência T2 de RMs no próprio software e mensuradas em milímetros (mm):

- Distância do EBC: a 15mm anterior à linha articular posterior à distância do eixo bicortical mediolateral femoral foi avaliada no nível do aparecimento dos dois epicôndilos femorais no corte axial de RM ( Fig. 1.1 ).
- DEM femoral: distância do ponto mais proximal e proeminente da crista do epicôndilo medial clínico até o ponto articular distal do côndilo femoral medial observado no corte coronal de RM ( Fig. 1.2 ).
- DEL femoral: distância do ponto mais proximal e proeminente do epicôndilo lateral da crista epicondilar até o ponto articular distal do côndilo femoral lateral observado no corte coronal de RM ( Fig. 1.3 ).

**Fig. 1:**
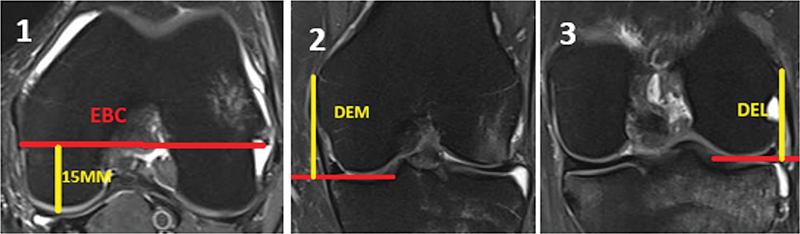
A Fig. 1.1 expressa a mensuração da distância do eixo bicortical condilar; a Fig. 1.2 demonstra a distância epicondilar medial à linha articular e a Fig.1.3 demonstra a distância epicondilar lateral à linha articular.

Todas as mensurações foram realizadas por um único cirurgião de joelho duas vezes e em dias diferentes. Foram computadas somente as mensurações que apresentaram variação < 10% na análise intraobservador.

As avaliações epidemiológicas como idade, sexo e distâncias morfológicas ósseas obtidas foram submetidas a análise estatística de média e desvio padrão (DP).

A metodologia de amostra e os cálculos das amostras foram determinados para um intervalo de confiança (IC) de 95%.

Para cada um dos 500 pares de valores da DEL *versus* EBC e DEM *versus* EBC, foram calculadas razões para o sexo masculino e feminino separadamente:



## Resultados

A média etária dos pacientes analisados foi de 50,91 ± 14,76 anos. A média de idade para o sexo masculino foi de 48,82 ± 15,43 anos e 53,01 ± 13,73 anos para o sexo feminino. A média de distância do EBC foi 72,11 ± 5,93mm, sendo 76,21 ± 4,83mm para os homens e 68,00 ± 3,64mm e para as mulheres. As DEM e DEL médias foram 33,39 ± 3,50mm e 26,32 ± 4,08mm, sendo para o sexo masculino de 35,20 ± 3,15mm e 27,11 ± 4,60mm e para o sexo feminino 31,58 ± 2,86mm e 25,53 ± 3,25mm ( Tabela 1 ).

**Tabela 1:** Dados epidemiológicos e antropomorfológicos dos joelhos com pacientes, idade, eixo bicortical condilar, distância epicôndilo medial à linha articular, e distância epicôndilo lateral à linha articular

	**Total (média ± DP)**	**Masculino (média ± DP)**	**Feminino (média ± DP)**
Pacientes	500	250	250
Idade (anos)	50,91 ± 14,76	48,82 ± 15,43	53,01 ± 13,73
EBC (mm)	72,11 ± 5,93	76,21 ± 4,83	68,00 ± 3,64
DEM (mm)	33,39 ± 3,50	35,20 ± 3,15	31,58 ± 2,86
DEL (mm)	26,32 ± 4,08	27,11 ± 4,60	25,53 ± 3,25

**Abreviações** : DEL, distância epicôndilo lateral à linha articular; DEM, distância epicôndilo medial à linha articular; DP, desvio padrão; EBC, eixo bicortical condilar.

A razão obtida no sexo feminino para o epicôndilo lateral foi de IC95% = 0,3720–0,3815 com margem de erro de ± 0,0048; já a razão obtida para o epicôndilo medial foi de IC95% = 0,4599–0,4707, com margem de erro de ± 0,0054. A razão obtida no sexo masculino para o epicôndilo lateral foi de IC95% = 0,3559–0,3671, com margem de erro de ± 0,0056, e para o epicôndilo medial foi de IC95% = 0,4568–0,4668, com margem de erro de ± 0,0050. Neste cenário, há evidências de que a verdadeira razão entre o epicôndilo lateral ou medial e a distância EBC está, com 95% de confiança, nos intervalos supracitados.

Desta forma, foram estabelecidas as seguintes fórmulas para o determinar a linha articular em relação aos EBC, DEM e DEL:



## Discussão

A relação das referências anatômicas epicondilares e distância biepicondilar do joelho como potencial de serem utilizadas na parametrização e determinação do posicionamento da linha articular femoral se apresentou satisfatória em joelhos da população brasileira com razões métricas adequadas em ambos os sexos.

Em procedimentos de ATJ de revisão, a manutenção da altura articular é uma condição fundamental para preservação da funcionalidade biomecânica do joelho no pós-operatório, de forma a garantir, com êxito, uma boa mobilidade e estabilidade articular do paciente. No entanto, as dificuldades técnicas para determinar esta referência anatômica é relevante devido às perdas ósseas. Quando o posicionamento da linha articular é sacrificado cirurgicamente, há um maior risco de comprometimento biomecânico articular e este fato está associado a desgaste, sobrecarga e instabilidade patelar seja por frouxidão ligamentar ou tensão excessiva, 8 9 10 sendo que a modificação de posicionamento da LA em 8mm está associada a alterações funcionais significativas e com piores desfechos clínicos de seguimento pós-operatório. 11 12

Neste sentido, foram descritos estudos que se propuseram a investigar a relação entre a linha articular e estruturas anatômicas como referência, a fim de estabelecer parâmetros intraoperatórios para se identificar a LA com os epicôndilos laterais e mediais, estruturas destacadas e fundamentais para tal propósito, demonstrando uma boa e precisa relação entre tais marcações anatômicas para esta finalidade. 3 4 Contudo, não identificamos estudos na literatura que buscaram estabelecer estes parâmetros específicos para a população brasileira.

Ressalta-se que diversas populações mundiais, como caucasianos, estado-unidenses, asiáticos e europeus têm apresentado resultados variáveis para os aspectos morfológicos dos joelhos, demonstrando que as características anatômicas podem divergir em algum grau das relações anatômicas de diversas populações étnicas, 6 13 14 15 16 e por estes achados é fundamental que populações miscigenadas, como a brasileira, tenha seus parâmetros articulares analisados para aumentar a confiança dos dados morfológicos articulares. A importância da realização de estudos em populações específicas se pauta na diversidade étnica mundial e em suas potenciais influências em características anatômicas populacionais. Desta forma, o presente estudo atribui aos cirurgiões brasileiros uma alternativa de aprimoramento de posicionamento do componente femoral, principalmente nas ATJ de revisão, a fim de contribuir com mais um potencial parâmetro de acurácia para se estimar a LA.

No presente estudo, optou-se por avaliar a relação do posicionamento da LA baseado em parâmetros anatômicos evidentes – epicôndilos durante uma ATJ de revisão em imagens de RM devido à maior facilidade para a identificação anatômica destes e mensurações das distâncias epicondilares se comparado a radiografias simples. A distância bicondilar é facilmente observada durante a cirurgia, ainda que os pontos proeminentes dos epicôndilos possam proporcionar alguma dificuldade de identificação acurada, ainda assim possível de se mapear intraoperatoriamente.

Os resultados encontrados demonstraram que é possível aplicar um multiplicador constante às distâncias EBC durante a cirurgia de ATJ com perdas ósseas a fim de se estimar, através dos epicôndilos mediais e ou laterais, o posicionamento da LA com IC95%, agregando maior acurácia e segurança ao cirurgião quando analisada a população brasileira. Observou-se que, em média, a DEM se apresentou mais proximal à DEL em 27% para o sexo masculino e em 23% para o sexo feminino. A existência de dois parâmetros de estimativa da LA baseado nos epicôndilos pode aumentar a acurácia cirúrgica para esta determinação, já que, a depender das características anatômicas de cada paciente e das dificuldades de acesso articular imputada pelas vias de acesso cirúrgicas, é possível que um determinado epicôndilo seja mais factível para identificação do que o outro.

A obtenção da posição da LA a partir do epicôndilo medial é um fato bem estudado e sedimentado na literatura vigente e sugere distâncias fixas entre 23mm e 35mm, dependendo das relações biotípicas dos pacientes. 3 4 17 Outros autores avaliaram o posicionamento da LA a partir da cabeça da fíbula e encontraram relação de 4mm a 22mm, sendo demonstrado que o parâmetro de menor variabilidade é a referência epicondilar quando comparada à cabeça da fíbula para a mesma finalidade. 3 4 18 19 Rajagopal et al. 8 estudaram a relação de proporcionalidade entre a distância interepicondilar e os epicôndilos até a superfície articular, que se demonstrou constante na população inglesa, concluindo que esta medida pode ser usada para prever a posição da altura da linha articular.

Os resultados do presente estudo possuem limitações e devem ser analisados com senso crítico pelo leitor. Não foram analisados os perfis biométricos de altura e peso dos pacientes a fim de compreender se as divergências anatômicas da população podem estar relacionadas a outras características biotípicas. Não foram analisados os perfis de miscigenação das amostras para determinar se os grupos estudados poderiam ter vieses de raça ou cor. Os cortes ósseos parametrizados no estudo radiológico podem não corresponder aos realizados por um cirurgião a depender das condições cirúrgicas, tais como hipoplasias ou defeitos ósseos, fato que poderia agregar viés de transposição dos resultados encontrados para estes casos.

## Conclusão

A distância do eixo bicortical condilar femoral pode ser usado como referência para a determinação do posicionamento da LA a partir das distâncias dos epicôndilos femorais medial e lateral.

## References

[1] Delanois R E Mistry J B Gwam C U Mohamed N S Choksi U S Mont M A Current Epidemiology of Revision Total Knee Arthroplasty in the United States J Arthroplasty 2017 32 09 2663 2668 10.1016/j.arth.2017.03.066 28456561

[2] Ferreira M C Oliveira J CP Zidan F F Franciozi C EDS Luzo M VM Abdalla R J Total knee and hip arthroplasty: the reality of assistance in Brazilian public health care Rev Bras Ortop 2018 53 04 432 440 10.1016/j.rboe.2018.05.002 30027075 PMC6052187

[3] Laskin R S Joint line position restoration during revision total knee replacement Clin Orthop Relat Res 2002 404 169 171 10.1097/00003086-200211000-00029 12439257

[4] Servien E Viskontas D Giuffrè B M Coolican M R Parker D A Reliability of bony landmarks for restoration of the joint line in revision knee arthroplasty Knee Surg Sports Traumatol Arthrosc 2008 16 03 263 269 10.1007/s00167-007-0449-y 18046537

[5] Han H Oh S Chang C B Kang S B Anthropometric difference of the knee on MRI according to gender and age groups Surg Radiol Anat 2016 38 02 203 211 10.1007/s00276-015-1536-2 26253858

[6] Ha C W Na S E The correctness of fit of current total knee prostheses compared with intra-operative anthropometric measurements in Korean knees J Bone Joint Surg Br 2012 94 05 638 641 10.1302/0301-620X.94B5.28824 22529083

[7] Uehara K Kadoya Y Kobayashi A Ohashi H Yamano Y Anthropometry of the proximal tibia to design a total knee prosthesis for the Japanese population J Arthroplasty 2002 17 08 1028 1032 10.1054/arth.2002.35790 12478514

[8] Rajagopal T S Nathwani D Can interepicondylar distance predict joint line position in primary and revision knee arthroplasty? Am J Orthop 2011 40 04 175 178 21731925

[9] Yoshii I Whiteside L A White S E Milliano M T Influence of prosthetic joint line position on knee kinematics and patellar position J Arthroplasty 1991 6 02 169 177 10.1016/s0883-5403(11)80013-6 1875209

[10] Martin J W Whiteside L A The influence of joint line position on knee stability after condylar knee arthroplasty Clin Orthop Relat Res 1990 259 146 156 2208849

[11] Figgie H E 3rd Goldberg V M Heiple K G Moller H S 3rd Gordon N H The influence of tibial-patellofemoral location on function of the knee in patients with the posterior stabilized condylar knee prosthesis J Bone Joint Surg Am 1986 68 07 1035 1040 3745240

[12] Partington P F Sawhney J Rorabeck C H Barrack R L Moore J Joint line restoration after revision total knee arthroplasty Clin Orthop Relat Res 1999 367 165 171 10.1097/00003086-199910000-00020 10546611

[13] Mahfouz M Abdel Fatah E E Bowers L S Scuderi G Three-dimensional morphology of the knee reveals ethnic differences Clin Orthop Relat Res 2012 470 01 172 185 10.1007/s11999-011-2089-2 21948324 PMC3237843

[14] Urabe K Mahoney O M Mabuchi K Itoman M Morphologic differences of the distal femur between Caucasian and Japanese women J Orthop Surg (Hong Kong) 2008 16 03 312 315 10.1177/230949900801600309 19126897

[15] McNamara C A Hanano A A Villa J M Huaman G M Patel P D Suarez J C Anthropometric Measurements of Knee Joints in the Hispanic Population J Arthroplasty 2018 33 08 2640 2646 10.1016/j.arth.2018.03.052 29691176

[16] Hussain F Abdul Kadir M R Zulkifly A H Anthropometric measurements of the human distal femur: a study of the adult Malay population Biomed Res Int 2013 2013 175056 10.1155/2013/175056 24294597 PMC3835611

[17] Stiehl J B Abbott B D Morphology of the transepicondylar axis and its application in primary and revision total knee arthroplasty J Arthroplasty 1995 10 06 785 789 10.1016/s0883-5403(05)80075-0 8749761

[18] Mason M Belisle A Bonutti P Kolisek F R Malkani A Masini M An accurate and reproducible method for locating the joint line during a revision total knee arthroplasty J Arthroplasty 2006 21 08 1147 1153 10.1016/j.arth.2005.08.028 17162174

[19] Tantavisut S Amarase C Ngarmukos S Tanavalee C Tanavalee A Knee joint line related to bony landmarks of the knee: a radiologic study in a Thai population Knee Surg Relat Res 2022 34 01 5 10.1186/s43019-022-00135-5 35168654 PMC8845375

